# A systematic review of multimorbidity patterns: social determinants and classification methods

**DOI:** 10.1093/eurpub/ckac130.048

**Published:** 2022-10-25

**Authors:** B Ramos-Fiol, ME Ortega-Martin, J Carretero-Bravo, V Suarez-Lledo, J Alvarez-Galvez

**Affiliations:** 1 Biomedicine, Biotechnology and Public Health, University of Cadiz, Jerez de la Frontera, Spain; 2 Clinical Medicine and Public Health, University of Granada, Granada, Spain

## Abstract

**Background:**

Governments and healthcare systems are facing multimorbidity (MM) as a major challenge due to the difficulties related to its proper identification and clinical management. Despite growing research on MM, its epidemiology is poorly understood due to the great complexity of underlying patterns of chronicity. The present review aims to identify the most frequent MM profiles and their social determinants.

**Methods:**

A systematic review following the PRISMA statement was conducted. The search strategy was performed by combining three sets of keywords (MM, inequalities and patterns) that were searched in Pubmed, Scopus, Web of Science, OVID, CINAHL Complete, and PsycINFO. Primary studies analysing MM patterns and their relationship with social determinants were included. The quality of the studies was assessed using the Axis tool quality assessment.

**Results:**

After the review process, 96 studies were selected from the 46,726 identified. The main methods used to identify MM patterns fell into five categories: latent class analysis (38.54%), cluster techniques (23.96%), factor analysis (19.79%), and machine learning (10.42%), and expert knowledge (7.29%). Latent class analysis was widely used, although in recent years the use of techniques based on machine learning has increased. The main patterns were cardiometabolic, cardiovascular, mental, musculoskeletal, complex MM, and respiratory diseases. Some MM profiles were more prevalent among lower-SES groups. In particular, patterns of mental multimorbidity were more prevalent among women and complex patterns were associated with low income.

**Conclusions:**

Results show different disease combinations among disparate social determinants such as gender, age, education, and socio-economic status. Our results suggest that more and better designed studies are needed to improve clinical practice and health policies with the aim of enhancing the quality of patients with MM and their relationship to health system use and care.

**Key messages:**

- Patterns of mental multimorbidity and complex multimorbidity were more prevalent among women and men of low socioeconomic status, respectively.
- An increasing number of studies are using a network-based approach to classify multimorbidity.

